# Mesenchymal stem cells‐derived and siRNAs‐encapsulated exosomes inhibit osteonecrosis of the femoral head

**DOI:** 10.1111/jcmm.15395

**Published:** 2020-08-04

**Authors:** Chi Zhang, Yan Su, Hao Ding, Jimin Yin, Zhenhong Zhu, Wenqi Song

**Affiliations:** ^1^ Department of Orthopaedics Shanghai Jiao Tong University Affiliated Sixth People's Hospital Shanghai China

**Keywords:** BMSCs, ONFH, siRNAs‐encapsulated BMSCexos

## Abstract

Osteonecrosis of the femoral head (ONFH) is a progressive, obstinate and disabling disease. At present, the treatment of ONFH is still a global medical problem. We aim to explore the role of bone mesenchymal stem cells (BMSCs)‐derived and siRNAs‐encapsulated exosomes (siRNAs‐encapsulated BMSCexos) in ONFH. We first isolated BMSCexos and screened siRNAs of 6 ONFH‐related genes for siRNAs‐encapsulated BMSCexo. The expression of these 6 ONFH‐related genes in dexamethasone (DXM)‐treated MC3T3‐E1 cell, cell model of ONFH, was detected by RT‐qPCR and Western blot analysis. And then, we performed CCK‐8 assay, angiogenesis assay and HE staining analysis to test the promotion role of the siRNAs‐encapsulated BMSCexo for angiogenesis during ONFH repair. The results suggest that the obtained particles were BMSCexos. The screened effective siRNAs could effectively knock down their expression in VECs. Moreover, siRNAs‐encapsulated BMSCexo could effectively knock down the expression of these genes in VECs. In addition, siRNAs‐encapsulated BMSCexo promote angiogenesis during ONFH repair. In conclusion, we found siRNAs‐encapsulated BMSCexos could promote ONFH repair by angiogenesis, and indicated exosome as the new siRNA carrier is of great significance to improve the efficiency of RNAi.

## 1 INTRODUCTION

Osteonecrosis of the femoral head (ONFH) is a progressive, obstinate and disabling disease. With the development of ONFH, the joint collapses could seriously affect the daily life and work of the patients. Moreover, only artificial hip replacement could be performed in the later stage of the disease, with the continuous expansion of osteonecrosis. But for young‐ and middle‐aged patients with artificial joint, that replacement will face treatment risks and difficulties later. So, how to effectively treat ONFH and prevent the further development of the disease has become the core research content of the treatment of ONFH. According to epidemiological reports, the incidence of ONFH is increasing year by year in the world.^1^, ^2^ The incidence of steroid‐induced ONFH accounts for more than half of non‐invasive ONFH. If not treated in time, 70% of the patients will eventually collapse the femoral head, which will bring heavy psychological and economic burden to individuals and society.

At present, the treatment of ONFH is still a global medical problem. How to maximize the recovery of patients' motor function has been a difficult issue in clinical research. In the past few decades, many researchers have tried various methods such as hip conserving therapy, stem cell transplantation, release of nutrition drugs and tissue engineering technology to repair the ONFH, but these treatment measures have disadvantages in clinical effect. Now, the methods of hip conserving operation in our hospital, such as core decompression, fibula transplantation with vascular pedicle, vascular bundle implantation and surface replacement, have achieved a certain effect. However, there are still some controversies on core decompression as a relief method of ONFH. The fibula transplantation with vascular pedicle is relatively mature, but the surgical trauma is relatively large. Therefore, it is an urgent task for clinicians and researchers to find a new effective and restorative treatment.

Stem cells have strong ability of proliferation and multi‐directional differentiation and have been widely used in the repair of many kinds of tissue damage.^3^, ^4^, ^5^ It has been proved that stem cells have the function of promoting osteoblasts and blood vessels.^6^, ^7^, ^8^ Recently, it is believed that after stem cells are transplanted to the injured tissue, tissue repair is not derived from the proliferation and differentiation of the implanted cells themselves, but from the paracrine function of stem cells.^9^, ^10^ Stem cells secrete growth factors, cytokines, chemokines, microbubbles and exosomes to the injured site, which promote the chemotaxis, proliferation and differentiation of the injured site cells. Among these secretions, exosomes play an important role in intercellular transport, signal transduction and tissue regeneration.^11^, ^12^, ^13^, ^14^

Exosomes have a lipid bilayer membrane structure, which could protect the coated substances well and could target specific cells or tissues,^15^, ^16^ as a messenger of communication between cells, to avoid immune exclusion. At present, the commonly used gene therapy vectors such as virus vectors and liposomes will inevitably stimulate the immune system response. Exosomes could carry protein miRNA, mRNA and DNA during cell communication. RNA interference (RNAi) is a kind of gene silencing technology which could shut down the expression of specific target genes. siRNA, as an exogenous synthetic RNA molecule, could play a role in cells through cell uptake or virus delivery. It has an important prospect in exploring the application of gene function and the direction of gene therapy. However, now, RNAi transport vectors are mainly viruses or synthetic reagents. These carriers are often highly toxic and low specific. Exosomes as siRNA transporters have not been reported before. In this study, we found that the bone mesenchymal stem cells‐derived and siRNAs‐encapsulated exosomes (BMSCexos), as the carrier of siRNA, could promote the repair of ONFH through angiogenesis, which could provide theoretical basis for the study of the repair of ONFH. At the same time, exosomes as a new siRNA carrier are of great significance to improve the efficiency of RNAi.

## 2 MATERIALS AND METHODS

### 2.1 Isolation of BMSCexos

BMSCs were isolated from C57BL/6 mice. Exos from the cell supernatants were isolated using the ExoQuick™ Plasma Prep and Exosome Precipitation Kit (SBI System Biosciences, San Francisco Bay Area, California, USA). BMSCs cells isolated from mice were cultured. When the confluence of cells reached 80%‐90%, the old medium was discarded and washed with PBS for three times, and the serum‐free medium was added. After 48 hours of culture, the supernatant was collected, and 3000 g centrifuged for 15 minutes to remove the cell fragments and the culture exfoliated cells. The supernatant was transferred to a new centrifuge tube, and the 0.5‐fold volume of exosomes was added to mix. After incubation at 4°C overnight, centrifugation was carried out at 4°C, 1500 g and 30 minutes under the condition of ultralow temperature centrifuge the next day. Discard the supernatant and add 100 μl PBS for heavy suspension and standby.

### 2.2 Cell culture

Cell lines HUVEC, HMEC‐1 and MC3T3‐E1 were purchased from CAS (Chinese Academy of Sciences, Shanghai, China). HUVEC and MC3T3‐E1 cells were cultured in DMEM medium (Gibco, Gaithersburg, MD, USA). HMEC‐1 cells were cultured in ECM medium (Gibco). Medium contained 10% foetal bovine serum (FBS), and all cells were cultured in a humidified atmosphere consisting of 5% CO_2_ and 95% air at 37°C.

### 2.3 Quantitative real‐time polymerase chain reaction (QRT‐PCR) assay

Total RNA was extracted from samples using TRIzol Reagent (Invitrogen, Carlsbad, California, USA). The cDNA was synthesized using Primescipt RT reagent kit with gRNA Eraser (TaKaRa, Dalian, Liaoning, China). QRT‐PCR was performed with SYBR® Premix Ex Taq™ II (Tli RNaseH Plus) (TaKaRa, Dalian, Liaoning, China).

### 2.4 Western blot

Western blot of samples was performed with ECL Western Blotting Substrate Kit (Abnova, Neihu, Taibei, China) and antibodies (Abcam, Cambridge Science Park, Cambridge, UK). Cells were subcultured. When the confluence of the cells reached 90% and the cells were in good condition, a single cell suspension was prepared. 2 × 105 cells/mL were inoculated into 6‐well plates, with a volume of 2 mL per well. After incubating for 24 hours at 37°C and 5% CO_2_ saturated humidity incubator, when the cell convergence reached 80%‐90%, exocrine treatment was performed. After 24 hours treatment, the cells were collected, extracted and quantified and then separated by 10% SDS‐polyacrylamide gel (120 V), electrophoretic (100 V, 120 minutes) to PVDF membrane. After the 5% BSA was sealed at room temperature for 1 hours, the membrane was incubated overnight at 4°C with the first antibody diluted at a ratio of 1:1000. TBST was rinsed three times, each time for 10 minutes. And then, the HRP‐labelled secondary antibody was incubated at room temperature for 1 hours. TBST was rinsed three times, each time for 10 minutes. ECL detection, exposure and development were performed after rinsing.

### 2.5 CCK‐8 assay

The proliferation ability of cells was evaluated using a CCK‐8 kit (Dojindo, Kumamoto, Kyushu, Japan) according to the manufacturer's instructions. Cells were subcultured. When the confluence of the cells reached 90% and the cells were in good condition, a single cell suspension was prepared. 2 × 10^5^ cells/mL were inoculated into 6‐well plates, with a volume of 2 mL per well. After 24 hours of culture in 37°C and 5% CO_2_ saturated humidity incubator, when the convergence degree of cells reaches 80%‐90%, exosomes are treated. After 24 hours of treatment, the supernatant was discarded, and 10% CCK‐8 culture medium was added to each hole and continued to culture for 2‐3 hours. The light absorption value of the septum was detected by the multi‐functional enzyme analyser, and the detection wavelength was 450 nm. Calculation formula: cell proliferation inhibition rate = (1 − (light absorption value of experimental group − light absorption value of blank control hole)/(light absorption value of control group − light absorption value of blank control hole)) × 100%.

### 2.6 Angiogenesis assay

The angiogenesis ability of cells was evaluated using Angiogenesis Assay Kit (BioVision, San Francisco Bay Area, California, USA). Angiogenesis was observed under an inverted microscope (Olympus, Tokyo, Japan). The cells were subcultured. When the confluence of the cells reached 90% and the cells were in good condition, a single cell suspension was prepared. 2 × 10^5^ cells/mL were inoculated into the 24‐well plate treated with matrix glue, and the volume of each well was 0.5 mL. After 24 hours culture in 37°C and 5% CO_2_ saturated humidity incubator, when the confluence of cells reaches 80%‐90%, exosomes are treated. After 24 hours treatment, the old medium was discarded, and each well was added with 500 µL (1:200) diluted staining solution and continued to be incubated in the incubator for 30 minutes and then observed by fluorescence microscope.

### 2.7 HE staining

HE staining analysis was performed with Hematoxylin and Eosin Staining Kit (Beyotime, Shanghai, China), and tissues were obtained from the methylprednisolone‐treated C57BL/6 mice, animal model of ONFH. The obtained bone tissues of mice were fixed with 4% paraformaldehyde and then decalcified with decalcification solution, embedded with paraffin and sliced with slicer. Each slice was 5 μm thick. The slices were stained with haematoxylin solution for 5‐10 minutes and then were washed and stained with eosin solution for 2 minutes. Next, the slices were treated with 70% ethanol for 10 seconds, 80% ethanol for 10 seconds, 90% ethanol for 10 seconds and anhydrous ethanol for 10 seconds. Xylene is transparent for 5 minutes. Change to fresh xylene and make it transparent for another 5 minutes. Seal with neutral gum or other sealer. Under the microscope, the nucleus is blue, while the cytoplasm is pink or red.

### 2.8 Statistical analyses

Statistical analyses were conducted with SPSS 21.0 statistical software (IBM, Armonk, NY, USA). The difference between two groups was analysed by *t* test and corrected by Welch. One‐way ANOVA was used for the data subject to normal distribution. *P* values <0.05 were considered statistically significant.

## 3 RESULTS

### 3.1 BMSCexos inhibit ONFH

**Figure 1:**
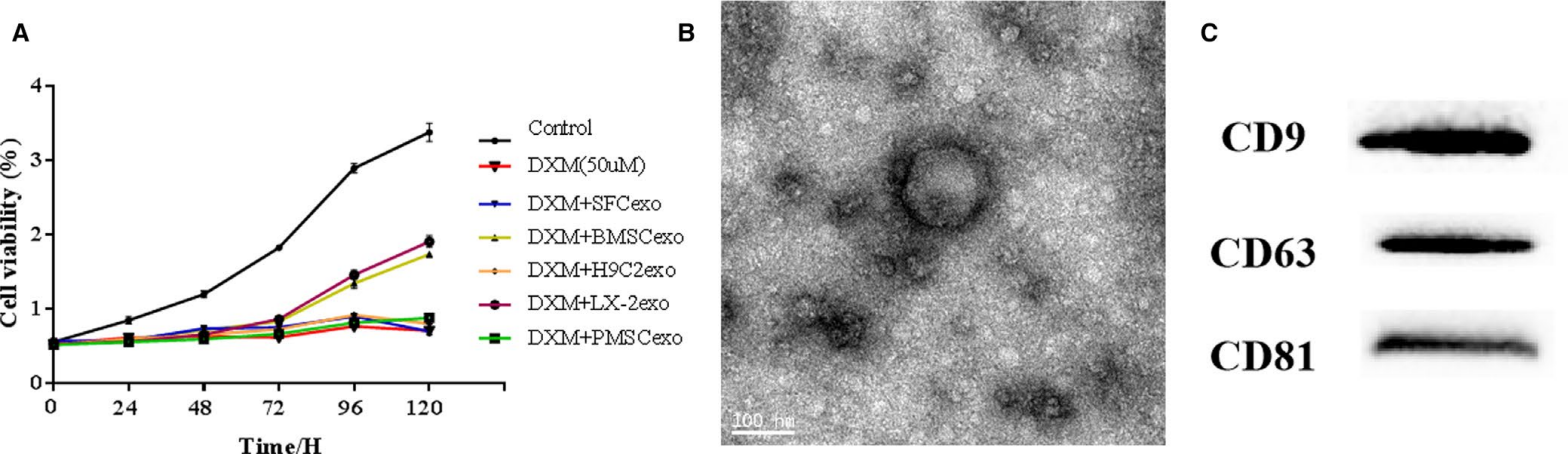
BMSCexos inhibit ONFH. (A) The repair effect of BMSCexo was better than other groups. (B) The morphology of BMSCexo was photographed by TEM (scale bar, 100 nm). (C) Western blotting of exosomal markers CD9, CD63 and CD81 in BMSCexo

The cell model of steroid‐induced ONFH was established by dexamethasone (DXM) ‐induced apoptosis of mouse embryonic osteoblast precursor cell MC3T3‐E1. DXM‐treated MC3T3‐E1 cells were treated with BMSCexo, hepatic stellate cell‐derived exosomes (LX‐2exo), cardiac cell‐derived exosomes (H9C2exo), placental mesenchymal stem cell‐derived exosomes (PMSCexo) and skin fibroblast‐derived exosomes (SFCexo), respectively, in an attempt to find a better therapeutic exosomes source. We performed CCK‐8 assay to detect the proliferation ability of VECs after co‐culture with these 5 kinds of exo, and results suggested that the repair effect of BMSCexo was better than that of other groups (Figure 1A). Exos were mainly evaluated based on their size, shape and specific proteins. TEM showed that the obtained particles from BMSCs were 100‐150 nm in size and appeared homogeneous with a complete membrane structure (Figure 1B). Western blotting revealed that the obtained particles significantly expressed exosomal markers CD9, CD63 and CD81 in BMSCexos (Figure 1C). Collectively, these results suggest that the obtained particles were BMSCexos.

### 3.2 siRNAs screening of ONFH‐related genes for siRNAs‐encapsulated BMSCexo

**Figure 2:**
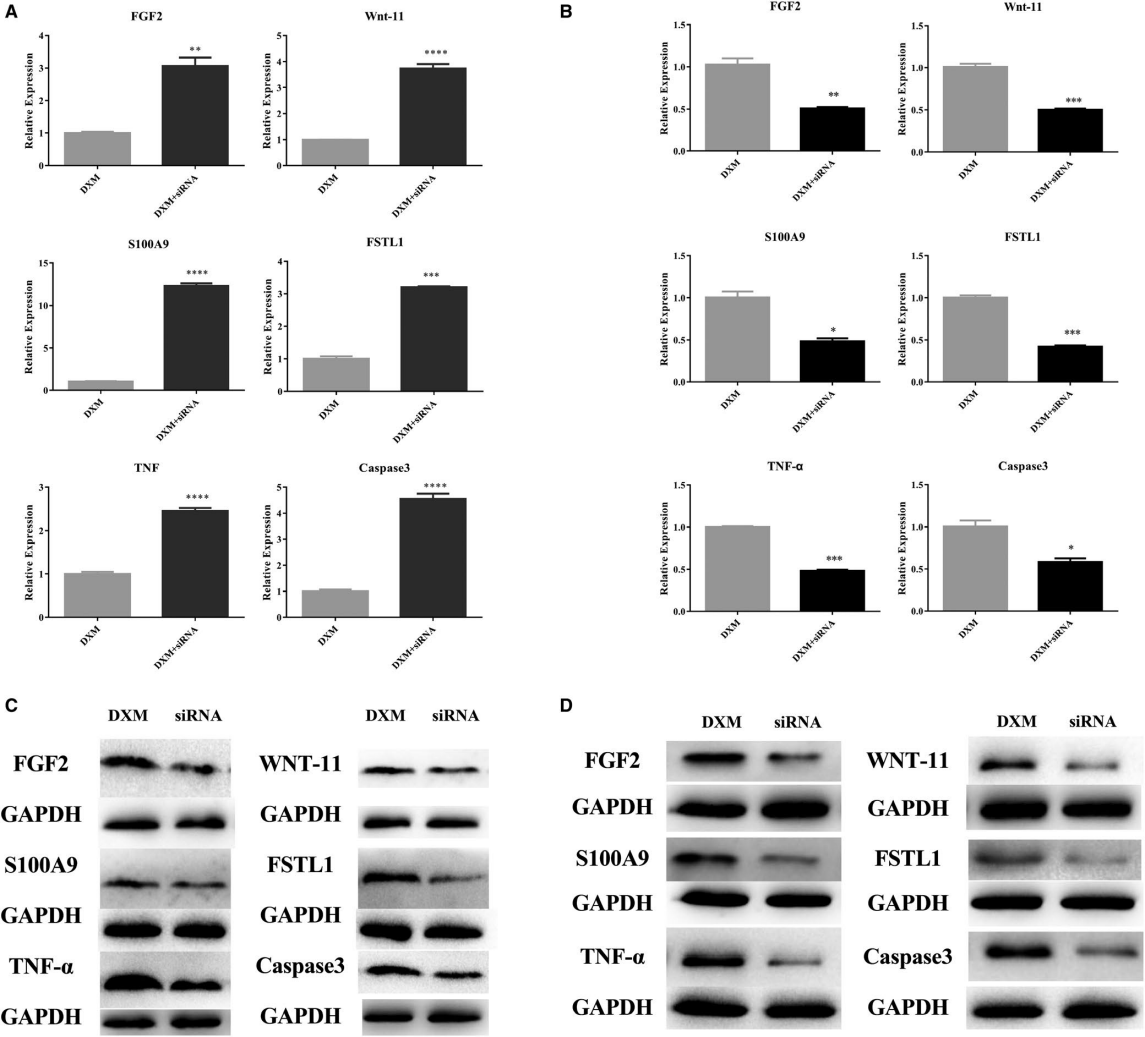
siRNAs screening of ONFH‐related genes for siRNAs‐encapsulated BMSCexo. (A) The expression of FGF2, Wnt‐11, S100A9, FSTL1, TNF‐α and Caspase3 in DXM‐treated MC3T3‐E1 cells was measured by RT‐qPCR. (B) The protein expression of FGF2, Wnt‐11, S100A9, FSTL1, TNF‐α and Caspase3 in DXM‐treated MC3T3‐E1 cells was detected by Western blot analysis. (C) RT‐qPCR measured the expression of FGF2, Wnt‐11, S100A9, FSTL1, TNF‐α and Caspase3 in VECs after transfected with each effective siRNA. (D) Western blot analysis detected the protein expression of FGF2, Wnt‐11, S100A9, FSTL1, TNF‐α and Caspase3 in VECs after transfected with each effective siRNA

6 ONFH‐related genes (FGF2, Wnt‐11, S100A9, FSTL1, TNF‐α and Caspase3), which have been already indicated high expressed in ONFH,^17^, ^18^, ^19^, ^20^, ^21^, ^22^ were selected for siRNAs screening. The expression of FGF2, Wnt‐11, S100A9, FSTL1, TNF‐α and Caspase3 in DXM‐treated MC3T3‐E1 cells, cell model of ONFH, was measured by RT‐qPCR (Figure 2A). And the protein expression of these 6 genes in DXM‐treated MC3T3‐E1 cells was detected by Western blot analysis (Figure 2B). Collectively, results confirmed these 6 genes were high expressed in ONFH. Next, we designed and synthesized siRNA interference fragments for these gene sequences and performed RT‐qPCR and Western blot analysis to detect the expression of them in vascular endothelial cells (VECs) after transfected with siRNA (Figure 2C,D). Therefore, we screened the effective siRNAs which could effectively knock down their expression in VECs.

### 3.3 Effective knock‐down of siRNAs‐encapsulated BMSCexo in VECs

**Figure 3:**
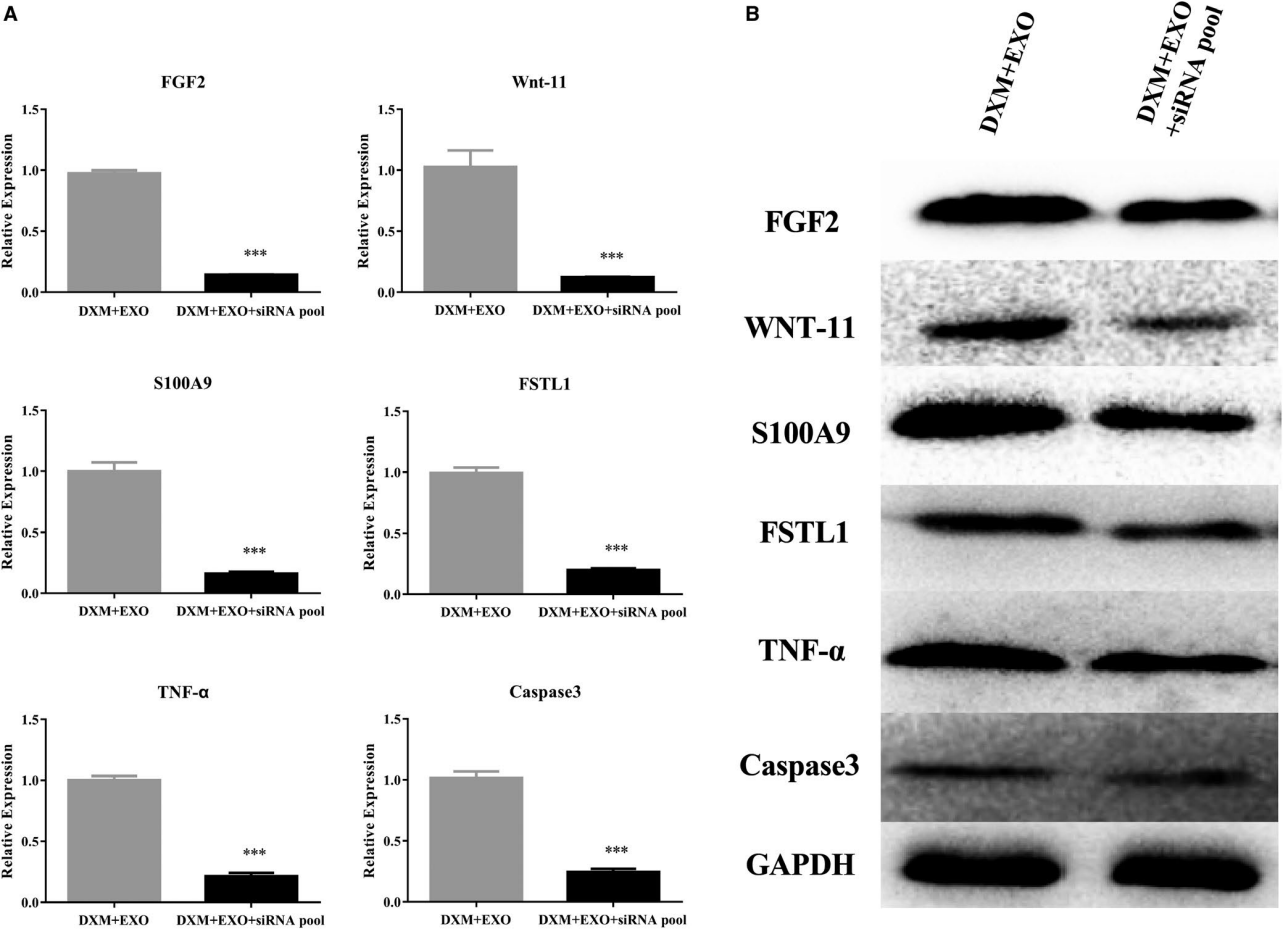
Effective knock‐down of siRNAs‐encapsulated BMSCexo in VECs. (A) The expression of FGF2, Wnt‐11, S100A9, FSTL1, TNF‐α and Caspase3 in VECs after co‐cultured with siRNA‐encapsulated BMSCexo measured by RT‐qPCR. (B) The protein expression of FGF2, Wnt‐11, S100A9, FSTL1, TNF‐α and Caspase3 in VECs after co‐cultured with siRNA‐encapsulated BMSCexo detected by Western blot analysis

We enclosed the screened effective siRNAs by BMSCexo in vitro and then detected the effective knock‐down of siRNAs‐encapsulated BMSCexo in VECs. The expression of FGF2, Wnt‐11, S100A9, FSTL1, TNF‐α and Caspase3 in VECs after co‐culture with siRNA‐encapsulated BMSCexo was measured by RT‐qPCR (Figure 3A). Moreover, the protein expression of them in VECs after co‐culture with siRNA‐encapsulated BMSCexo was detected by Western blot analysis (Figure 3B). Collectively, these data suggest siRNAs‐encapsulated BMSCexo could effectively knock down the expression of these genes in VECs.

### 3.4 siRNAs‐encapsulated BMSCexo promotes angiogenesis during ONFH repair

**Figure 4:**
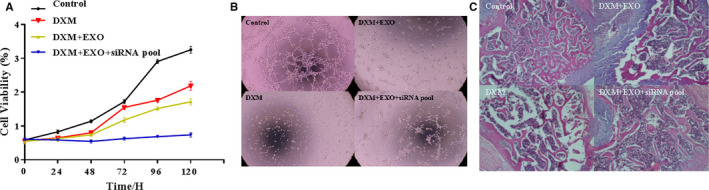
siRNAs‐encapsulated BMSCexo promotes angiogenesis during ONFH repair. (A) CCK‐8 assay. siRNA‐encapsulated BMSCexo significantly increased the proliferative ability of VECs in a dose‐dependent manner. (B) The ability of angiogenesis in VECs (× 100). (C) HE staining analysis. More bone trabecular tissue and fewer empty lacunae were observed in the siRNAs‐encapsulated BMSCexo group compared with the BMSCexo group

We performed CCK‐8 assay to detect the proliferative ability of vascular endothelial cells (VECs) after co‐cultured with the siRNA‐encapsulated BMSCexo. And results showed siRNA‐encapsulated BMSCexo could significantly increase the proliferative ability of vascular endothelial cells (VECs) than BMSCexo (Figure 4A). The ability of angiogenesis was also detected in VECs (×100) (Figure 4B). Moreover, HE staining analysis was performed after the necrotic tissues were obtained from methylprednisolone‐treated C57BL/6 mice, animal model of ONFH, and results showed that more bone trabecular tissue and fewer empty lacunae were observed in the siRNAs‐encapsulated BMSCexo group compared with the BMSCexo group (Figure 4C).

## 4 DISCUSSION

In this study, we found BMSCexo could promote ONFH repair through angiogenesis. And indicated it could serve as the new siRNA carrier to improve the efficiency of RNAi transfection. Exosome is ideal system for siRNA encapsulation,^23^, ^24^, ^25^, ^26^ because its structure is a hollow aqueous core, which is enveloped by phospholipid bilayer. Exosomes have good stability in the blood and natural targeting properties for delivering functional RNA into cells.^27^ Moreover, Alvarez‐Erviti *et al* had successfully demonstrated siRNA could be effective delivered to mice brains using engineered exosomes with virtually no complications.^26^ It is suggested that exosome‐based therapy is relatively safer than other therapies as exosomes do not replicate endogenously as cells would.^23^ Therefore, our data provided theoretical basis for the study of the ONFH repair, but its clinical application needs further exploration.

Exosomes could be isolated from cell culture and physiological fluids. The most popular method is using ultracentrifugation to pellet exosomes from materials.^26^, ^27^, ^28^ This method usually co‐precipitates proteins from the sample and could be always harsh on exosomes. For reducing protein and non‐exosomal contamination from the isolated exosomes, combining density‐based separation and ultracentrifugation is becoming more and more common.^24^, ^29^ Although size‐exclusion chromatography (SEC) could allow the exosomes separation from other types of extracellular vesicles (EV) by size, it is limited by only processing small amount of starting material^30^, ^31^ and that could also result in minimal protein contamination. Furthermore, immunoaffinity capture, which uses antibodies‐coated beads to bind to exosomal surface proteins, could allow specific capture of exosomes rather than EVs and could also isolate sub‐population of exosomes from samples. However, it is too costly and could also only process small amount of starting material.^31^, ^32^ Moreover, polymer‐based precipitation, which is a rather popular method in the past, is very crude for exosomes precipitation and it could lead to protein and non‐exosomal vesicle contamination.^33^, ^34^

Electroporation has been reported to load exosomes with siRNA, but with protein aggregation.^23^, ^25^, ^26^ Transfection‐based approaches also have been demonstrated to have better loading efficiency and protein stability, but it is undesirable because of its toxicity and side effects.^25^ Thus, as electroporation is a safer method, it has been more widely used in siRNA loading into exosomes. However, an optimized encapsulation method needs to be established in order to deliver adequate amounts of siRNA to the target site for a potent gene knock‐down. Therefore, exosomes from BMSCs could be isolated and subsequently loaded with various therapeutic siRNA for different types of target genes overexpressed in ONFH. Moreover, a clinical application about siRNAs‐encapsulated exosome needs to be further explored. And it should be translated to animal models to assess the efficiency of both the delivery and therapeutic efficiency of siRNA‐encapsulated exosome in vivo.

In conclusion, our research successfully demonstrated the encapsulation of siRNA into exosomes and their subsequent intracellular delivery of the siRNA to cells in vitro. We found BMSCexos could promote ONFH repair through angiogenesis. Our data provided theoretical basis for the study of ONFH repair, and it is of great significance to improve the efficiency of RNAi.

## CONFLICT OF INTEREST

The authors confirm that there are no conflicts of interest.

## AUTHORS’ CONTRIBUTION

Chi Zhang, Yan Su and Wenqi Song designed the research study and performed the research; Hao Ding contributed essential reagents or tools; Hao Ding and Jimin Yin analysed the data; Zhenhong Zhu and Wenqi Song wrote the paper.

## Data Availability

Research data are not shared.
